# Novel compound heterozygous *WDR35* variants in a Chinese patient associated with cranioectodermal dysplasia and ectopic testis: a case report and review of the literature

**DOI:** 10.1186/s12887-023-04110-1

**Published:** 2023-08-18

**Authors:** Lijie Li, Cuihua Liu, Ming Tian, Guangbo Li, Jitong Li

**Affiliations:** 1 Department of Nephrology and Rheumatology, Zhengzhou Key Laboratory of Pediatric Kidney Disease Research, Children’s Hospital Affiliated to Zhengzhou University, Henan Children’s Hospital, Zhengzhou Children’s Hospital, Zhengzhou, 450018 China; 2 Henan Provincial Key Laboratory of Children’s Genetics and Metabolic Diseases, Zhengzhou, 450018 China

**Keywords:** *WDR35*, Cranioectodermal dysplasia, Ciliopathy, Ectopic testis

## Abstract

**Background:**

*WDR35* variants are known to cause a rare autosomal recessive disorder-Cranioectodermal dysplasia (CED). The CED patients are commonly present with facial dysmorphisms (frontal bossing and low-set ears), sagittal craniosynostosis, growth retardation, dolichocephaly, skeletal deformities (brachydactyly, terminal hypoplasia of the fingers and narrow thorax), ectodermal abnormalities (sparse hair, and finger/toe nail dysplasia), nephronophthisis, retinal dystrophy and hepatic fibrosis. Diagnosis of CED can be difficult because it presents with high genetic heterogeneity. However, our understanding of the phenotype of CED caused by *WDR35* variants could be more explicit, and the correlation between genotype and phenotype needs further improvement.

**Case presentation:**

We report a case of the first Chinses patient of CED caused by *WDR35* variants, a 3-year-and-3-month-old patient, who was admitted to our hospital with frontal bossing, growth retardation, low set ears, dolichocephaly, sparse hair, and small limbs, abnormal renal function, and moderate anemia. The child showed a novel phenotype of the ectopic testis except for presenting typical CED characteristics, and he was identified with novel compound heterozygous *WDR35* variants (c.2590 C > T, p.Gln864* and c.2408_2416del, p.Asn803_Ala805del; NM_001006657). He was given iron succinate and erythropoietin to improve anemia and to inhibit repeated metabolic acidosis and hyperkalemia through acid correction, diuretic, and potassium-lowering treatments. The parents refused to accept renal replacement therapy for their child and were discharged voluntarily.

**Conclusions:**

This is the first reported case of the *WDR35* variants that can lead to CED and ectopic testis, which is also the first Chinese patient associated with *WDR35* variants. This study expands our understanding of genotype-phenotype association in patients with *WDR35* variants and provides genetic counseling for prevention and intervention in this genetic disorder. Neonatal carriers should be followed up for kidney and CED-related diseases to detect warning signs.

**Supplementary Information:**

The online version contains supplementary material available at 10.1186/s12887-023-04110-1.

## Background

Cranioectodermal dysplasia (CED), also known as Sensenbrenner syndrome, is a rare autosomal recessive disorder with high genetic heterogeneity in clinical manifestations [1]. Its primary clinical features include sagittal craniosynostosis, dolichocephaly, ectodermal abnormalities (sparse hair, hypodontia/microdontia and finger/toe nail dysplasia), skeletal deformities (narrow thorax, brachydactyly and terminal hypoplasia of the fingers), characteristic facial features (frontal bossing and low-set ears), growth retardation and joint laxity. The visceral anomalies include nephronophthisis, retinal dystrophy and hepatic fibrosis [2, 3]. In contrast, liver and kidney function are the main factors determining the CED prognosis [4]. Six genes, including *IFT122, WDR35, IFT43, WDR19, IFT52*, and *IFT140*, have been identified to be associated with the disease, and the *WDR35* variants are one of the most common causes of CED patients [5, 6].

The diagnosis of CED is usually based on the patient’s clinical characteristics and imaging results, as well as the identification of pathogenic genes through genetic testing [2]. Its clinical symptoms are diverse and may vary between families and intrafamilial members [6]. Therefore, it is particularly essential to pay close attention to the clinical characteristics of patients and conduct early genetic screening for the early diagnosis of CED patients.

Although 41 families have been diagnosed with CED through genetic testing, there is still a lack of knowledge about their genotypes and phenotypes [2]. In this study, we report the affected individual of a Chinese 3-year-and-3-month-old patient presenting with abnormal renal function, moderate anemia, frontal bossing, low set ears, growth retardation, sagittal craniosynostosis, dolichocephaly, narrow thorax, sparse hair, and small limbs. Moreover, we found this patient’s novel clinical manifestation of the ectopic testis. Molecular analysis showed that he carried novel compound heterozygous variants of a nonsense variant (c.2590 C > T, p.Gln864*) and a deletion variant (c.2408_2416del, p.Asn803_Ala805del) in *WDR35*. We describe the detailed clinical characteristics of one Chinese patient and present a novel clinical feature that might be associated with CED, which further expands the genotype-phenotype spectrum of *WDR35* and provides help to early precision diagnoses and genetic counseling.

## Case presentation

A 3-year-and-3-month-old boy presented with abnormal renal function, frontal bossing, low-set ears, dolichocephaly, sparse hair, and short limbs. He was the seventh child of his unrelated parents and was delivered naturally at full term with a birth weight of 3.8 kg. He could walk at the age of 1 year and 3 months and has language retardation. On admission, a physical examination showed that the patient was developmentally delayed, with longer skull diameter, macrocephaly, sparse and delicate hair, narrow eye cleft, hypertelorism, low bridge of the nose, sparse teeth with dysplasia, short and curved fingers/toes, and the testes were not touched in the bilateral scrotum.

**Fig. 1:**
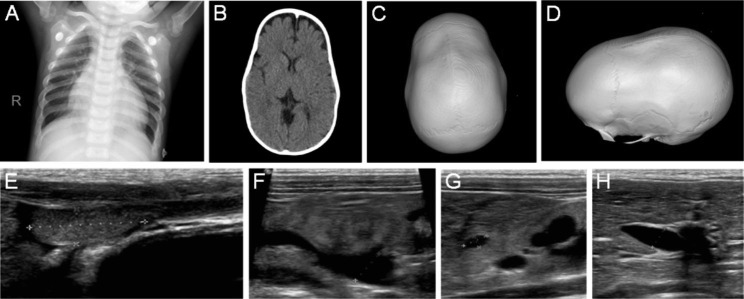
The patient at the age of 3 years and 3 months. **a** Frontal chest radiographs showed increased heart volume, decreased bone density, and enlarged trabeculae; **b** CT scan showed the bilateral frontocephalic space widened; **c-d** the anteroposterior diameter of the skull increased, and the sagittal suture was partially closed in three-dimensional images; **e** abdominal color ultrasound examination showed the right testicle located in the iliac fossa, **f** the liver volume was increased, the parenchymal echo was dense and enhanced, and the intrahepatic bile duct was widened, **g** the intrahepatic hypoechoic mass was enlarged, **h** enhanced kidney parenchymal echo, unclear demarcation of cortex and medulla, and hydronephrosis of the right kidney

Computed tomography (CT) examination of the head revealed that the bilateral frontocephalic space had widened, the anteroposterior diameter of the skull had increased, and the sagittal suture was partially closed. Frontal chest radiographs showed increased heart volume, decreased bone density, and enlarged trabeculae. Electrocardiogram and color echocardiography were normal. The abdominal color ultrasound examination revealed that the liver volume was increased, the parenchymal echo was dense and enhanced, the intrahepatic hypoechoic mass was enlarged, and the intrahepatic bile duct was widened. Dysplasia of both kidneys enhanced parenchymal echo, unclear demarcation of the cortex and medulla, hydronephrosis of the right kidney and ureter, and dilatation of the left calyces. The spleen is enlarged with open sinuses. In particular, the child had an abnormal testicle position, with the right testicle located in the iliac fossa and the left testicle situated in the groin, and a hydrocele existed on both sides (Fig. 1A-H). These were not found in the previously reported CED patients.

The biochemical indicators of the patient showed that the urea value was 28.3 mmol/L (reference range: 1.7–7.1 mmol/L), the creatinine value was 432.1 umol/L (reference range: 18–80 umol/L), and the uric acid value was 393.8 umol/L (reference range: 134–415 umol/L). The 24-hour (h) urine volume was only 350 ml (reference range: 300-1500ml), and the 24 h urine protein quantity was 0.416 g (reference range: 0.03–0.15 g/24 h). Urine-specific gravity is 1.010 (reference range: 1.015–1.025), and pH is 7.0 (reference range: 5.5–6.5). The urinary protein and creatinine ratio was 4.32 mg/mmol (reference range: ≤0.2 mg/mmol), urinary microalbumin was 595.0 mg/L (reference range: 0–20 mg/L), α1-microglobulin was 39.56 mg/L (reference range: 0–12 mg/L), urinary transferrin was 2.52 mg/L (reference range:<1 mg/L), and urinary β2- microglobulin was 422.70 ug/L (reference range: 0-300 ug/L). His parathyroid hormone level was 739.400 pg/mL (reference range: 15–65 pg/mL). These data indicate that the child’s renal function is significantly abnormal. Moreover, routine blood tests showed that the value of erythrocytes was 2.28 × 10^12^/L (reference range: 3.5–5.5 × 10^12^/L) and the hemoglobin value was 65 g/L (reference range: 120–170 g/L), suggesting moderate anemia.

**Fig. 2:**
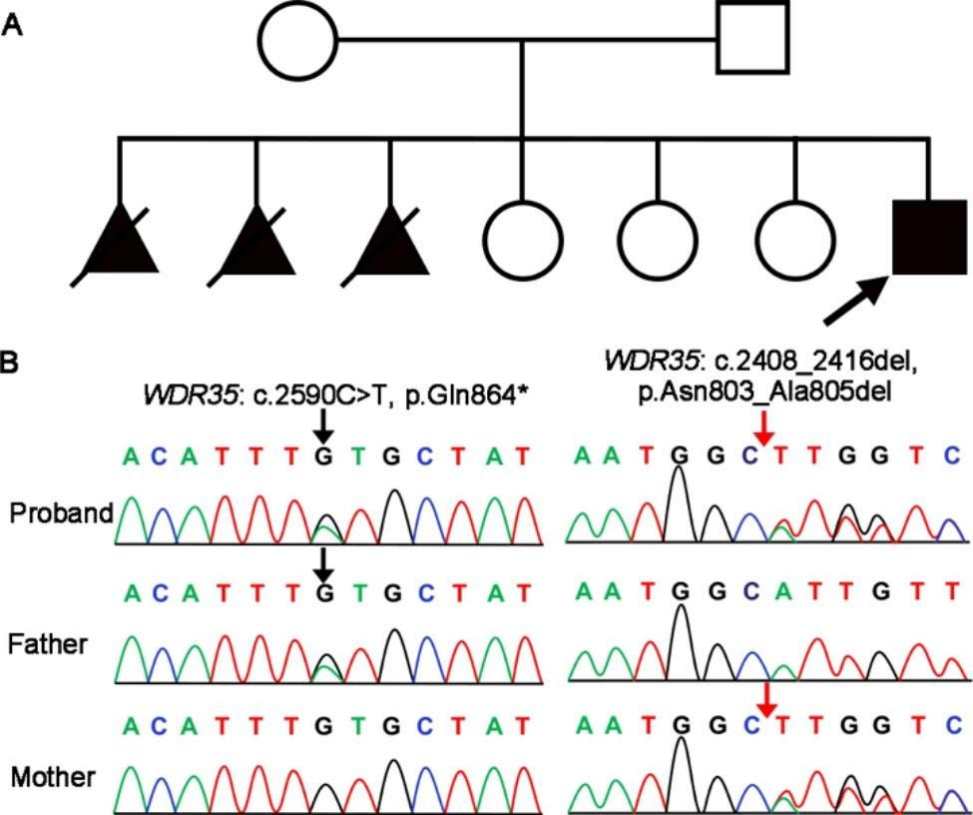
Family pedigree and genetic testing. **a** Family pedigree of the CED patient, with the proband indicated by a black arrow, and the aborted fetuses of anonymous sex are shown as black triangles with oblique lines. **b** Sanger sequencing chromatograms of the two variants in *WDR35* from the patient and his parents. Capital letters represent the genomic sequences. The patient carries a novel compound heterozygous variant of *WDR35* for the c.2590 C > T substitution (black arrow) and c.2408_2416del in-frame variant (red arrow)

The patient had no family history and no genetic disease or nephropathy among his family members. He has three healthy sisters, and his mother has experienced three miscarriages (Fig. 2A). Because the child has a unique appearance and multi-system and multi-organ abnormalities of the body, we suspect he has a genetic disease. With the informed consent of the boy and his parents, we performed a trio-whole exome sequencing (trio-WES) for them and verified the results by Sanger sequencing. It was found that the patient carried a c.2590 C > T (p.Gln864*) nonsense variant and a c.2408_2416del (p.Asn803_Ala805del) in-frame variant of the *WDR35* gene (NM_001006657), which were derived from his father and mother, respectively (Fig. 2B).

**Fig. 3:**
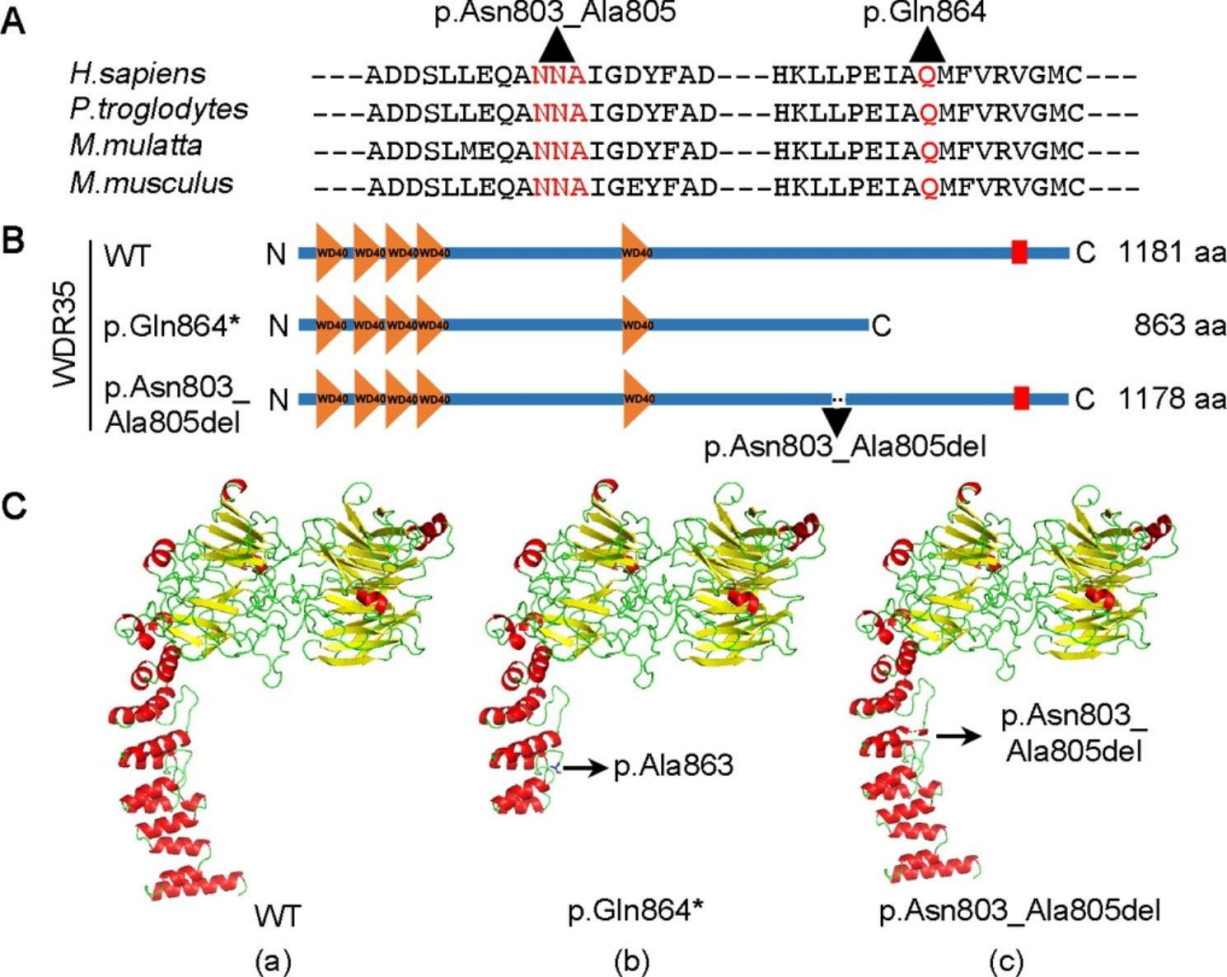
Analysis of the conservation, plain and crystal structures of the WDR35 domains using bioinformatic tools. **a** Alignment of the amino acid sequences of human WDR35 (NM_001006657) with the WDR35 sequences of chimpanzee (XM_009442060.3), rhesus monkey (XM_001107482.4) and mouse (NM_001159527.1). The p.Asn803_Ala805 and p.Gln864 amino acids are indicated in red. **b** Schematic representation of the effects of WDR35 variants on protein structure. The p.Gln864* variant in WDR35 caused the truncation for 318 amino acids at its C-terminal. In comparison, the p.Asn803_Ala805del variants led to the loss of three amino acids and the in-frame variant. WT, wild type; orange-colored triangles indicate WD40 repeat regions; red boxes represent low-complexity structural domain; aa, amino acids. **c** Cartoon structural models of human WDR35 domains designed with the SWISS-MODEL tool and visualized with PyMOL software. We used yeast β’-COP (3mkq.1.A) to construct human WDR35 crystal structures based on the high homology of their protein sequences. (a) The normal WDR35 protein structure with α-helix in red, β-fold in yellow and an irregular curl in green; (b) the truncated p.Gln864* protein of WDR35 only harboring the residual 863 amino acids; (c) the p.Asn803_Ala805del mutant protein missing three amino acids at the position 803_805

To our knowledge, this is the first CED patient caused by *WDR35* variants in the Chinese population. Moreover, the child presented with a novel phenotype of ectopic testis except for showing typical CED characteristics. The patient carries a novel compound heterozygous variant that includes a c.2590 C > T nonsense variant and a c.2408_2416del in-frame variant derived from his father and mother, respectively. According to the American College of Medical Genetics and Genomics (ACMG) guidelines, these two variants were classified as “pathogenic” and “uncertain” variants. A comparison of the amino acid sequences of WDR35 proteins from different species showed that p.Gln864 and p.Asn803_Ala805 are highly conserved across species (Fig. 3A), suggesting that amino acid sequences at these sites play a vital role in the function of this protein. The p.Gln864* variant can lead to a truncation of 318 amino acids at C-terminal, and the p.Asn803_Ala805 deletion causes the loss of three amino acids and generates an in-frame mutant protein (Fig. 3A-B). Using the same bioinformatic tools reported previously [7], we analyzed the crystal structures of the mutant WDR35 protein by a SWISS-MODEL database with homology modeling using yeast β’-COP (3mkq.1.A) (Fig. 3C). The results showed that the crystal structure is truncated due to the p.Gln864* variant, which resulted in the disorder of the 3-dimensional system (Fig. 3C). This generates a loss of function of the protein. Alanine consistently stabilizes the helical conformation because it buries a more apolar area upon folding and because its backbone entropy is lower [8], thus its loss might cause the altered conformation of the protein. Asparagine-linked (*N*-linked) glycosylation is one of the most complex enzyme-catalyzed protein modification reactions. The modification can influence early events in protein folding, mediating significant effects locally, on the adjacent polypeptide sequence, and at remote sites distant from the modified asparagine residue [9]. Therefore, we speculate that the compound heterozygous variants in *WDR35* may lead to the loss of function of the protein, which is pathogenic for the patient.

The child was given iron succinate and erythropoietin to improve anemia after a routine blood examination after admission. He had repeated metabolic acidosis and hyperkalemia and was given acid correction, diuretic, and potassium-lowering treatments. However, the parents refused further treatment using renal replacement therapy for the child and were discharged voluntarily. The patient died when followed up six months later.

## Discussion and conclusions

*WDR35* and *IFT122* variants are the most common causes of CED, accounting for approximately 60% of CED families [10]. WDR35/IFT121 is involved in the assembly of the IFT-A complex and functions as a subunit of IFT-A in retrograde transport to the ciliary base [6, 11]. Moreover, it is essential for cilia assembly, playing a role in the Rab8 vesicle development of nascent cilia by selecting different transport cargoes involved in ciliary protein excretion and plays a crucial role in the centripetal satellite organization [12]. Thus, CED is also a type of ciliopathy. Cilia are organelles on the apical surface of cells in almost all tissues and organs. They integrate multiple signaling pathways essential for vertebrates’ development and organ differentiation. Therefore, cilia dysfunction can cause various human diseases [13].

By reviewing the literature, we analyzed the genotype-phenotype association of *WDR35*, and the results showed that the missense variant of *WDR35* is the primary type of variation. Moreover, *WDR35* variants can induce various diseases or syndromes, including CED, Nephronophthisis, Short rib-polydactyly syndrome, Cleft lip and/or palate, Periventricular nodular heterotopia, Autism, Ductal plate malformation, Ellis-van Creveld syndrome and Jeune asphyxiating thoracic dystrophy. Among them, CED is the most common syndrome caused by *WDR35* variants. Craniosynostosis is one of the characteristic manifestations of CED patients, and existing studies have shown that 80% of patients have craniosynostosis [1]. However, our analysis of the clinical phenotypes associated with the *WDR35* variants revealed that only 40% of the patients had this manifestation. Additionally, the clinical characteristics are not the same for the same genotype, whether within or among the patients’ families. Here we found the patient had ectopic testicular manifestation except for having the typical CED features, which were not found in the previous reports. An additional table file shows these in more detail [see Additional file 1]. The above results indicate that CED patients have high genetic heterogeneity, and the analysis of the clinical characteristics of different pathogenic genes and the genotype-phenotype association of the same pathogenic gene is of great importance for the accurate diagnosis and early treatment of such patients.

Primary cilia are linearly arranged on the polarized epithelial cells surrounding the renal tubules involved in signaling pathways that control directed cell division and are essential in developing renal tubules. Moreover, the relationship between primary cilia and cystic kidney disease has also been demonstrated in a study of *Caenorhabditis elegans* [14]. In this paper, the CED patient is also accompanied by bilateral renal dysplasia, right renal and ureteral hydronephrosis, left calyx dilatation, and abnormal renal function, which might be related to the dysfunction of primary cilia. Additionally, we found the patient presented with ectopic testicles, a novel feature in CED syndrome. Some patients with Bardet-Biedl syndrome (BBS) also showed an ectopic testicular phenomenon [15, 16], which verifies the possible overlap of different ciliopathy phenotypes. Sloboda et al. reported that the occurrence of ectopic testes in patients with BBS was related to fetal hypogonadism [15], and WDR35 and BBS proteins have similar functions in the transport of vesicles in cilia. Therefore, we speculate that the ectopic testes of the patient reported in this study may also be related to fetal gonadal dysfunction, but this needs further investigation. Moreover, RNA sequencing (RNA-seq) results of normal human tissues showed relatively high expression of *WDR35* in the testis [17], which further proved that *WDR35* might play an essential role in the genesis and function maintenance of the testis. Therefore, the occurrence of ectopic testicles in the patient in this study is likely caused by the *WDR35* variants, which further enriches the genotype and phenotype spectrum of CED resulting from *WDR35* variants.

In conclusion, we report the first Chinese CED child identified with a novel compound heterozygous variant in *WDR35* (c.2590 C > T, c.2408_2416del). Moreover, we found the patient presented with a novel phenotype of the ectopic testis except for showing the typical CED characteristics. Our case report can expand our understanding of genotype-phenotype association in patients with *WDR35* variants and provide genetic counseling for prevention and intervention in this genetic disorder.

### Electronic supplementary material

Below is the link to the electronic supplementary material.

Supplementary Material 1

## Data Availability

The datasets used in this case are available from the corresponding author on reasonable request.

## Abbreviations

BBS: Bardet-Biedl syndrome
CED: Cranioectodermal dysplasia
CT: Computed tomography
h: Hour
RNA-seq: RNA sequencing
WES: Whole exome sequencing
