# Supplementary material for: Novel compound heterozygous WDR35 variants in a Chinese patient associated with cranioectodermal dysplasia and ectopic testis: a case report and review of the literature
Source: BMC Pediatr. 2023 Aug 18;23:407. doi: 10.1186/s12887-023-04110-1 (PMC10436638; doi:10.1186/s12887-023-04110-1)
Supplement: Supplementary file 1 — Supplementary Material 1 [file 12887_2023_4110_MOESM1_ESM.docx]

**Table S1.** Analysis of the genotype-phenotype association of patients with *WDR35* variants

| **Patie-nt ID** | **Sex** | **Nucleot-ide change** | **Amino acid change** | **Growth retardatio-n** | **Dolich-cephaly** | **Cranios-ynostosi-s** | **Spars-e hair** | **Ophthal-mologi-cal problem-s** | **Low set ears** | **Dyspl-astic teeth** | **Narro-w thorax** | **Short limbs** | **Inguina-l hernia** | **Renal disease** | **Hepatic disease** | **Lung diseas-e** | | **Nervou-s system disease** | **Heart defect** | **Reference** |  |
| --- | --- | --- | --- | --- | --- | --- | --- | --- | --- | --- | --- | --- | --- | --- | --- | --- | --- | --- | --- | --- | --- |
|  |  |  |  |  |  |  |  |  |  |  |  |  |  |  |  |  |  |  |  |  |  |
| 1 | NA | c.1877A>G | p.Glu626Gly | + | + | + | - | + | + | + | + | + | + | - | - | + | - | | - | [1] |  |
|  |  | c.25-2A>G | p.Ile9Thrfs*7 |  |  |  |  |  |  |  |  |  |  |  |  |  |  |  |  |  |  |
| 2 | NA | c.2891delT | p.Pro964Leufs*15 | + | + | + | + | - | + | + | + | + | + | - | - | - | - | | - | [1] |  |
|  |  | c.2623G>A | p.Ala875Thr |  |  |  |  |  |  |  |  |  |  |  |  |  |  |  |  |  |  |
| 3 | Female | c.622G>C | p.Ala208Pro | + | - | - | - | + | - | - | - | - | - | + | - | - | + | | - | [2] |  |
|  |  | c.2134delC | p.Leu712Tyrfs*20 |  |  |  |  |  |  |  |  |  |  |  |  |  |  |  |  |  |  |
| 4 | NA | c.1433G>A | p.Arg478Lys | - | - | - | - | - | + | - | + | + | - | + | - | - | - | | - | [3] |  |
|  |  | c.1579C>T | p.Gln527* |  |  |  |  |  |  |  |  |  |  |  |  |  |  |  |  |  |  |
| 5 | NA | c.932G>T | p.Trp311Leu | - | - | - | - | NA | - | - | + | + | - | NA | - | - | NA | | - | [3] |  |
|  |  | c.1501delC | p.Gln501Lysfs*10 |  |  |  |  |  |  |  |  |  |  |  |  |  |  |  |  |  |  |
| 6 | Male | c.2891delT | p.Pro964Leufs*15 | - | - | - | - | - | - | - | + | + | - | + | NA | - | - | | + | [4] |  |
|  |  | c.2623G>A | p.Ala875Thr |  |  |  |  |  |  |  |  |  |  |  |  |  |  |  |  |  |  |
| 7 | Female | c.2891delT | p.P964Lfs*15 | - | - | - | - | - | - | - | + | - | - | + | - | - | - | | - | [4] |  |
|  |  | c.2623G>A | p.Ala875Thr |  |  |  |  |  |  |  |  |  |  |  |  |  |  |  |  |  |  |
| 8 | Female | c.932G>T | p.Trp311Leu | - | - | - | - | + | - | + | - | - | - | + | + | + | - | | - | [4] |  |
|  |  | c.3396-29_3396-18del | NA |  |  |  |  |  |  |  |  |  |  |  |  |  |  |  |  |  |  |
| 9^#^ | Male | c.1592T>C | p.Leu531Pro | - | + | + | - | + | + | - | + | + | - | + | + | - | + | | + | [5] |  |
|  |  | c.1592T>C | p.Leu531Pro |  |  |  |  |  |  |  |  |  |  |  |  |  |  |  |  |  |  |
| 10^#^ | Female | c.1592T>C | p.Leu531Pro | - | - | - | - | - | - | - | + | + | - | + | + | - | + | | + | [5] |  |
|  |  | c.1592T>C | p.Leu531Pro |  |  |  |  |  |  |  |  |  |  |  |  |  |  |  |  |  |  |
| 11^#^ | Fetuses | c.1592T>C | p.Leu531Pro | - | NA | NA | NA | NA | NA | NA | NA | NA | NA | NA | NA | NA | NA | | NA | [5] |  |
|  |  | c.1592T>C | p.Leu531Pro |  |  |  |  |  |  |  |  |  |  |  |  |  |  |  |  |  |  |
| 12 | Male | c.1592T>C | p.Leu531Pro | + | + | - | - | - | + | - | + | - | - | - | - | - | - | | + | [5] |  |
|  |  | c.1592T>C | p.Leu531Pro |  |  |  |  |  |  |  |  |  |  |  |  |  |  |  |  |  |  |
| 13 | Female | c.2912A>G | p.Tyr971Cys | + | + | + | + | - | + | - | + | + | - | - | - | - | + | | - | [6] |  |
|  |  | c.2912A>G | p.Tyr971Cys |  |  |  |  |  |  |  |  |  |  |  |  |  |  |  |  |  |  |
| 14 | Female | c.504T>A | p.Ser168Arg | + | + | + | + | + | + | + | - | + | - | + | + | - | + | | - | [6] |  |
|  |  | c.1922T>G | p.Leu641* |  |  |  |  |  |  |  |  |  |  |  |  |  |  |  |  |  |  |
| 15 | Male | c.3091C>T | p.His1031Tyr | + | - | + | + | - | + | - | - | + | - | - | - | - | + | | - | [7] |  |
|  |  | c.3203A>G | p.Tyr1068Cys |  |  |  |  |  |  |  |  |  |  |  |  |  |  |  |  |  |  |
| 16 | Male | c.707G>A | p.Cys236Tyr | + | + | - | - | + | + | - | + | + | - | + | - | + | + | | + | [8] |  |
|  |  | c.1922T>G | p.Leu641* |  |  |  |  |  |  |  |  |  |  |  |  |  |  |  |  |  |  |
| 17 | NA | c.2161C>T | NA | NA | NA | NA | NA | NA | NA | NA | NA | NA | NA | NA | NA | NA | NA | | NA | [9] |  |
|  |  | NA |  |  |  |  |  |  |  |  |  |  |  |  |  |  |  |  |  |  |  |
| 18 | Male | c.3459G>T | p.Trp1153Cys | + | - | - | - | - | - | + | + | + | - | + | + | - | - | | - | [10] |  |
|  |  | c.3459G>T | p.Trp1153Cys |  |  |  |  |  |  |  |  |  |  |  |  |  |  |  |  |  |  |
| 19 | NA | c.932G>T | p.Trp311Leu | - | - | - | - | - | + | - | + | + | - | - | - | - | NA | | - | [11] |  |
|  |  | - | NA |  |  |  |  |  |  |  |  |  |  |  |  |  |  |  |  |  |  |
| 20 | Female | c.206G>A | p.Gly69Asp | - | - | - | - | + | - | - | - | - | - | + | + | - | + | | - | [12] |  |
|  |  | c.206G>A | p.Gly69Asp |  |  |  |  |  |  |  |  |  |  |  |  |  |  |  |  |  |  |
| 21 | Female | c.206G>A | p.Gly69Asp | - | - | - | - | + | - | - | - | - | - | + | + | - | + | | - | [12] |  |
|  |  | c.206G>A | p.Gly69Asp |  |  |  |  |  |  |  |  |  |  |  |  |  |  |  |  |  |  |
| 22 | Female | c.1922T>G | p.Leu641* | + | + | + | + | + | + | + | + | + | - | + | - | - | - | | - | [13] |  |
|  |  | c.2522A>T | p.Asp841Val |  |  |  |  |  |  |  |  |  |  |  |  |  |  |  |  |  |  |
| 23^#^ | Female | c.1922T>G | p.Leu641* | + | + | + | - | - | + | - | + | + | - | + | + | + | - | | - | [13] |  |
|  |  | c.2522A>T | p.Asp841Val |  |  |  |  |  |  |  |  |  |  |  |  |  |  |  |  |  |  |
| 24 | Male | c.1415G>A | p.Arg472Gln | + | + | - | + | + | - | + | + | - | - | - | - | + | + | | - | [14] |  |
|  |  | c.1415G>A | p.Arg472Gln |  |  |  |  |  |  |  |  |  |  |  |  |  |  |  |  |  |  |
| 25 | Male | c.337C>T | p.Arg113* | - | - | + | + | - | + | + | + | + | - | + | - | - | + | | + | [15] |  |
|  |  | c.2522A>T | p.Asp841Val |  |  |  |  |  |  |  |  |  |  |  |  |  |  |  |  |  |  |
| 26 | NA | c.1433+3A>G | NA | NA | NA | NA | NA | NA | NA | NA | NA | NA | NA | NA | NA | NA | NA | | NA | [16] |  |
|  |  | c.932G>T | p.Trp311Leu |  |  |  |  |  |  |  |  |  |  |  |  |  |  |  |  |  |  |
| 27 | NA | c.1922T>G | p.Leu641* | NA | NA | NA | NA | NA | NA | NA | NA | NA | NA | NA | NA | NA | NA | | NA | [16] |  |
|  |  | c.2522A>T | p.Asp841Val |  |  |  |  |  |  |  |  |  |  |  |  |  |  |  |  |  |  |
| 28 | NA | c.1922T>G | p.Leu641* | NA | NA | NA | NA | NA | NA | NA | NA | NA | NA | NA | NA | NA | NA | | NA | [16] |  |
|  |  | c.2522A>T | p.Asp841Val |  |  |  |  |  |  |  |  |  |  |  |  |  |  |  |  |  |  |
| 29 | Male | c.3G>A | p.Met1_Ala30delinsMetfs*4 | + | + | + | + | - | + | + | + | + | + | + | - | - | - | | - | [17] |  |
|  |  | c.2522A>T | p.Asp841Val |  |  |  |  |  |  |  |  |  |  |  |  |  |  |  |  |  |  |
| 30 | Male | c.3G>A | p.Met1_Ala30delinsMetfs*4 | + | + | + | + | - | + | + | + | + | + | + | - | - | - | | - | [17] |  |
|  |  | c.2522A>T | p.Asp841Val |  |  |  |  |  |  |  |  |  |  |  |  |  |  |  |  |  |  |
| 31 | Male | c.1633C>T | p.Arg545* | + | - | - | - | - | - | - | - | - | - | + | + | - | + | | - | [18] |  |
|  |  | c.308G>T | p.Gly103Val |  |  |  |  |  |  |  |  |  |  |  |  |  |  |  |  |  |  |
| 32 | Male | c.2023C>T | p.Arg675* | + | + | + | - | + | - | - | - | + | - | + | - | - | + | | - | [19] |  |
|  |  | c.3378G>A | p.Leu1126Leu |  |  |  |  |  |  |  |  |  |  |  |  |  |  |  |  |  |  |
| 33 | Female | c.2023C>T | p.Arg675* | + | - | - | - | + | + | - | - | + | - | + | - | - | + | | - | [19] |  |
|  |  | c.3378G>A | p.Leu1126Leu |  |  |  |  |  |  |  |  |  |  |  |  |  |  |  |  |  |  |
| 34 | Female | c.994C>T | p.Arg332* | + | - | + | + | - | - | + | + | + | - | + | - | - | - | | - | [19] |  |
|  |  | c.2623G>A | p.Ala875Thr |  |  |  |  |  |  |  |  |  |  |  |  |  |  |  |  |  |  |
| 35 | NA | c.143-18T>A | NA | - | - | - | + | - | + | - | + | + | + | + | - | + | + | | - | [20] |  |
|  |  | c.143-18T>A |  |  |  |  |  |  |  |  |  |  |  |  |  |  |  |  |  |  |  |
| 36 | NA | c.143-18T>A | NA | - | - | - | + | - | - | - | + | - | - | - | - | + | + | | + | [20] |  |
|  |  | c.143-18T>A |  |  |  |  |  |  |  |  |  |  |  |  |  |  |  |  |  |  |  |
| 37 | Male | c.1194+1G>A | NA | + | - | - | - | - | - | + | - | - | - | + | + | - | - | | - | [20] |  |
|  |  | c.3378G>A |  |  |  |  |  |  |  |  |  |  |  |  |  |  |  |  |  |  |  |
| 38 | Female | c.1194+1G>A | NA | + | - | - | - | - | - | + | - | + | - | + | - | - | - | | - | [20] |  |
|  |  | c.3378G>A |  |  |  |  |  |  |  |  |  |  |  |  |  |  |  |  |  |  |  |
| 39 | Female | c.1434-684G>T | NA | + | + | - | + | - | - | - | + | + | - | + | - | - | - | | - | [20] |  |
|  |  | c.1434-684G>T |  |  |  |  |  |  |  |  |  |  |  |  |  |  |  |  |  |  |  |
| 40^*^ | Male | c.1922T>G | p.Leu641* | + | + | - | - | - | + | + | + | - | - | + | - | - | - | | - | [21] |  |
|  |  | c.2522A>T | p.Asp841Val |  |  |  |  |  |  |  |  |  |  |  |  |  |  |  |  |  |  |
| 41^*^ | Male | c.1922T>G | p.Leu641* | + | + | - | - | - | + | + | + | - | - | - | - | - | - | | - | [21] |  |
|  |  | c.2522A>T | p.Asp841Val |  |  |  |  |  |  |  |  |  |  |  |  |  |  |  |  |  |  |
| 42 | Female | c.206G>A | p.Gly69Asp | + | + | + | - | - | - | + | - | - | - | + | + | - | + | | + | [22] |  |
|  |  | c.206G>A | p.Gly69Asp |  |  |  |  |  |  |  |  |  |  |  |  |  |  |  |  |  |  |
| 43 | Male | - | - | + | + | + | - | - | - | - | - | - | - | + | + | - | + | | + | [23] |  |
|  |  | - | - |  |  |  |  |  |  |  |  |  |  |  |  |  |  |  |  |  |  |
| 44 | Male | c.1922T>G | p.Leu641* | + | + | - | + | - | + | + | + | + | + | + | - | + | + | | + | [24] |  |
|  |  | c.2522A>T | p.Asp841Val |  |  |  |  |  |  |  |  |  |  |  |  |  |  |  |  |  |  |
| 45 | Male | c.1922T>G | p.Leu641* | + | + | + | + | - | + | NA | + | + | + | + | + | NA | NA | | - | [24] |  |
|  |  | c.2522A>T | p.Asp841Val |  |  |  |  |  |  |  |  |  |  |  |  |  |  |  |  |  |  |
| 46 | Female | c.1922T>G | p.Leu641* | + | + | + | + | - | + | + | + | + | - | + | - | + | - | | - | [24] |  |
|  |  | c.2522A>T | p.Asp841Val |  |  |  |  |  |  |  |  |  |  |  |  |  |  |  |  |  |  |
| 47 | Male | c.2623G>A | p.Ala875Thr | - | - | - | - | + | + | + | - | - | + | + | - | - | - | | - | [25] |  |
|  |  | c.2907G>T | p.Lys969Asn |  |  |  |  |  |  |  |  |  |  |  |  |  |  |  |  |  |  |
| 48 | Female | c.206G>A | p.Gly69Asp | + | - | - | + | - | + | + | + | + | - | + | + | - | - | | - | [26] |  |
|  |  | c.206G>A | p.Gly69Asp |  |  |  |  |  |  |  |  |  |  |  |  |  |  |  |  |  |  |
| 49 | Male | c.907G>A | p.Gly303Arg | + | + | + | + | + | + | - | + | + | - | + | + | - | - | | - | [27] |  |
|  |  | c.1922 T>G | p.Leu641* |  |  |  |  |  |  |  |  |  |  |  |  |  |  |  |  |  |  |

Abbreviations: NA, not applicable.

# The pound sign indicates that the patient has died and no genetic analysis was performed.

* The asterisk represents a patient who underwent next-generation sequencing without confirmation via Sanger sequencing.

References

1. Gilissen C, Arts HH, Hoischen A, Spruijt L, Mans DA, Arts P, van Lier B, Steehouwer M, van Reeuwijk J, Kant SG *et al*: Exome sequencing identifies WDR35 variants involved in Sensenbrenner syndrome. *Am J Hum Genet* 2010, 87(3):418-423.

2. Yamamura T, Morisada N, Nozu K, Minamikawa S, Ishimori S, Toyoshima D, Ninchoji T, Yasui M, Taniguchi-Ikeda M, Morioka I *et al*: Rare renal ciliopathies in non-consanguineous families that were identified by targeted resequencing. *Clin Exp Nephrol* 2017, 21(1):136-142.

3. Duran I, Taylor SP, Zhang W, Martin J, Qureshi F, Jacques SM, Wallerstein R, Lachman RS, Nickerson DA, Bamshad M *et al*: Mutations in IFT-A satellite core component genes IFT43 and IFT121 produce short rib polydactyly syndrome with distinctive campomelia. *Cilia* 2017, 6:7.

4. Stokman MF, van der Zwaag B, van de Kar NCAJ, van Haelst MM, van Eerde AM, van der Heijden JW, Kroes HY, Ippel E, Schulp AJA, van Gassen KL *et al*: Clinical and genetic analyses of a Dutch cohort of 40 patients with a nephronophthisis-related ciliopathy. *Pediatric Nephrology* 2018, 33(10):1701-1712.

5. Bacino CA, Dhar SU, Brunetti-Pierri N, Lee B, Bonnen PE: WDR35 mutation in siblings with Sensenbrenner syndrome: a ciliopathy with variable phenotype. *Am J Med Genet A* 2012, 158A(11):2917-2924.

6. Hoffer JL, Fryssira H, Konstantinidou AE, Ropers HH, Tzschach A: Novel WDR35 mutations in patients with cranioectodermal dysplasia (Sensenbrenner syndrome). *Clin Genet* 2013, 83(1):92-95.

7. Lin AE, Traum AZ, Sahai I, Keppler-Noreuil K, Kukolich MK, Adam MP, Westra SJ, Arts HH: Sensenbrenner syndrome (Cranioectodermal dysplasia): clinical and molecular analyses of 39 patients including two new patients. *Am J Med Genet A* 2013, 161A(11):2762-2776.

8. Li Y, Garrod AS, Madan-Khetarpal S, Sreedher G, McGuire M, Yagi H, Klena NT, Gabriel GC, Khalifa O, Zahid M *et al*: Respiratory motile cilia dysfunction in a patient with cranioectodermal dysplasia. *Am J Med Genet A* 2015, 167A(9):2188-2196.

9. Pengelly RJ, Arias L, Martinez J, Upstill-Goddard R, Seaby EG, Gibson J, Ennis S, Collins A, Briceno I: Deleterious coding variants in multi-case families with non-syndromic cleft lip and/or palate phenotypes. *Sci Rep* 2016, 6:30457.

10. Smith C, Lamont RE, Wade A, Bernier FP, Parboosingh JS, Innes AM: A relatively mild skeletal ciliopathy phenotype consistent with cranioectodermal dysplasia is associated with a homozygous nonsynonymous mutation in WDR35. *Am J Med Genet A* 2016, 170(3):760-765.

11. Toriyama M, Lee C, Taylor SP, Duran I, Cohn DH, Bruel AL, Tabler JM, Drew K, Kelly MR, Kim S *et al*: The ciliopathy-associated CPLANE proteins direct basal body recruitment of intraflagellar transport machinery. *Nat Genet* 2016, 48(6):648-656.

12. Shaheen R, Patel N, Shamseldin H, Alzahrani F, Al-Yamany R, A AL, Ewida N, Anazi S, Alnemer M, Elsheikh M *et al*: Accelerating matchmaking of novel dysmorphology syndromes through clinical and genomic characterization of a large cohort. *Genet Med* 2016, 18(7):686-695.

13. Walczak-Sztulpa J, Wawrocka A, Sobierajewicz A, Kuszel L, Zawadzki J, Grenda R, Swiader-Lesniak A, Kocyla-Karczmarewicz B, Wnuk A, Latos-Bielenska A *et al*: Intrafamilial phenotypic variability in a Polish family with Sensenbrenner syndrome and biallelic WDR35 mutations. *Am J Med Genet A* 2017, 173(5):1364-1368.

14. Cordova-Fletes C, Becerra-Solano LE, Rangel-Sosa MM, Rivas-Estilla AM, Alberto Galan-Huerta K, Ortiz-Lopez R, Rojas-Martinez A, Juarez-Vazquez CI, Garcia-Ortiz JE: Uncommon runs of homozygosity disclose homozygous missense mutations in two ciliopathy-related genes (SPAG17 and WDR35) in a patient with multiple brain and skeletal anomalies. *Eur J Med Genet* 2018, 61(3):161-167.

15. Walczak-Sztulpa J, Wawrocka A, Swiader-Lesniak A, Socha M, Jamsheer A, Drozdz D, Latos-Bielenska A, Zachwieja K: Clinical and molecular genetic characterization of a male patient with Sensenbrenner syndrome (cranioectodermal dysplasia) and biallelic WDR35 mutations. *Birth Defects Res* 2018, 110(4):376-381.

16. Zhang W, Taylor SP, Ennis HA, Forlenza KN, Duran I, Li B, Sanchez JAO, Nevarez L, Nickerson DA, Bamshad M *et al*: Expanding the genetic architecture and phenotypic spectrum in the skeletal ciliopathies. *Hum Mutat* 2018, 39(1):152-166.

17. Walczak-Sztulpa J, Wawrocka A, Leszczynska B, Mikulska B, Arts HH, Bukowska-Olech E, Daniel M, Krawczynski MR, Latos-Bielenska A, Obersztyn E: Prenatal genetic diagnosis of cranioectodermal dysplasia in a Polish family with compound heterozygous variants in WDR35. *Am J Med Genet A* 2020, 182(10):2417-2425.

18. Strong A, Li D, Mentch F, Bedoukian E, Hartung EA, Meyers K, Skraban C, Wen J, Medne L, Glessner J *et al*: Ciliopathies: Coloring outside of the lines. *Am J Med Genet A* 2021, 185(3):687-694.

19. Quinaux T, Custodi V, Putoux A, Bacchetta J, Rossi M, Di Rocco F: Sensenbrenner syndrome: a further challenge in evaluating sagittal synostosis and a need for a multidisciplinary approach. *Childs Nerv Syst* 2021, 37(5):1695-1701.

20. Caparros-Martin JA, De Luca A, Cartault F, Aglan M, Temtamy S, Otaify GA, Mehrez M, Valencia M, Vazquez L, Alessandri JL *et al*: Specific variants in WDR35 cause a distinctive form of Ellis-van Creveld syndrome by disrupting the recruitment of the EvC complex and SMO into the cilium. *Hum Mol Genet* 2015, 24(14):4126-4137.

21. Brndiarova M, Mraz M, Kolkova Z, Cisarik F, Banovcin P: Sensenbrenner Syndrome Presenting with Severe Anorexia, Failure to Thrive, Chronic Kidney Disease and Angel-Shaped Middle Phalanges in Two Siblings. *Mol Syndromol* 2021, 12(4):263-267.

22. Antony D, Nampoory N, Bacchelli C, Melhem M, Wu K, James CT, Beales PL, Hubank M, Thomas D, Mashankar A *et al*: Exome sequencing for the differential diagnosis of ciliary chondrodysplasias: Example of a WDR35 mutation case and review of the literature. *Eur J Med Genet* 2017, 60(12):658-666.

23. Ackah RL, Yoeli D, Kueht M, Galvan NTN, Cotton RT, Rana A, O'Mahony CA, Goss JA: Orthotopic liver transplantation for Sensenbrenner syndrome. *Pediatr Transplant* 2018, 22(1).

24. Walczak-Sztulpa J, Wawrocka A, Stanczyk M, Pesz K, Dudarewicz L, Chrul S, Bukowska-Olech E, Wieczorek-Cichecka N, Arts HH, Oud MM *et al*: Interfamilial clinical variability in four Polish families with cranioectodermal dysplasia and identical compound heterozygous variants in WDR35. *Am J Med Genet A* 2021, 185(4):1195-1203.

25. Kaynar K, Kayipmaz S, Cebi AH, Huseynova S: Having Multiple Renal Cysts in a Young Adult is not Always a Sign of Polycystic Kidney Disease. *Balkan J Med Genet* 2021, 24(2):83-87.

26. Al Noaim K, Alfadhel M, Carre A, Polak M, Al Mutair A: Resolved Severe Primary Hypothyroidism in Sensenbrenner Syndrome Post Hepatorenal Transplantation: A Case Report. *Horm Res Paediatr* 2022.

27. Walczak-Sztulpa J, Wawrocka A, Sikora W, Pawlak M, Bukowska-Olech E, Kopaczewski B, Urzykowska A, Arts HH, Gotz-Wieckowska A, Grenda R *et al*: WDR35 variants in a cranioectodermal dysplasia patient with early onset end-stage renal disease and retinal dystrophy. *Am J Med Genet A* 2022, 188(10):3071-3077.
